# MITF maintains genome stability in nonmelanocyte lineages

**DOI:** 10.1002/1878-0261.70273

**Published:** 2026-06-10

**Authors:** Drifa H. Gudmundsdottir, Adrián López García de Lomana, Thejus B. Venkatesh, Kritika Kirty, Snaevar Sigurdsson, Linda Vidarsdottir, Ramile Dilshat, Erla Sveinbjornsdottir, Snaedis Ragnarsdottir, Daniel H Magnusson, Maria R. Bustos, Eirikur Steingrimsson, Thorkell Gudjonsson, Stefan Sigurdsson

**Affiliations:** ^1^ Cancer Research Laboratory, Faculty of Medicine, University of Iceland Reykjavik Iceland; ^2^ Biomedical Center, University of Iceland Reykjavik Iceland; ^3^ Department of Molecular Oncology University Hospital of Iceland Reykjavik Iceland; ^4^ Department of Genetics and Molecular Medicine University Hospital of Iceland Reykjavik Iceland; ^5^ Biotech Research & Innovation Center, University of Copenhagen Denmark

**Keywords:** genome instability, MITF, replication stress

## Abstract

Microphthalmia‐associated transcription factor (MITF) is crucial for development and survival of melanocytes and serves as a lineage‐specific oncogene that is amplified in 10–20% of melanomas. The role of MITF in pathways maintaining genome integrity, such as DNA replication, DNA repair, and mitosis has been extensively studied in melanocytes. In addition to its pro‐survival role in melanoma, recent studies have shown that MITF expression has important implications for cancer progression and treatment in other cancer types. Nevertheless, studies on the role of MITF in other tissues are scarce. Here, we show that depletion of MITF causes genome instability in nonmelanocytic cell lineages, which results in activation of P53, cell cycle arrest, and apoptosis. Moreover, we show that P53 activation in MITF‐depleted cells is dependent on LATS2, a kinase with an established role in the Hippo pathway. Finally, we show that LATS2 mediated upregulation of P53 is ATR‐dependent. Collectively, this study highlights the role of MITF as a genome maintenance factor beyond the melanocyte lineage, which might contribute to the tumor suppressive function of MITF.

## Graphical abstract

MITF is essential for melanocyte survival and acts as an oncogene in 10%–20% of melanomas. We show that MITF depletion causes genome instability in nonmelanocytic cells, leading to LATS2‐mediated P53 activation, cell cycle arrest, and apoptosis. This study highlights the role of MITF as a genome maintenance factor beyond the melanocyte lineage. Created in BioRender. Gudmundsdottir, D. H. (2026) https://BioRender.com/xcfqz2m

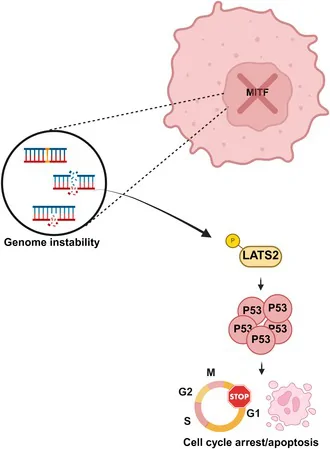

**Abbreviations**
(7AAD): 7‐aminoactinomycin D
(Aph): Aphidicolin
(BABs): Bulky anaphase bridges
(DEGs): Differentially expressed genes
(DSB): DNA double strand break
(HPA): The Human Protein Atlas
(LATS2): *Large tumor suppressor kinase 2*
(MDM2): *Mouse double minute 2 homolog*
(MITF): Microphthalmia‐associated transcription factor
(RPE): Retinal pigment epithelium
(TCGA): The Cancer Genome Atlas
(urDNA): Under‐replicated DNA

## 1 Introduction

MITF has an established role as a master regulator of melanocytes and a driver of melanoma progression [1, 2, 3]. Importantly, MITF controls plasticity in melanoma cells by switching between proliferating and metastatic states [4, 5]. Increased MITF expression has been associated with poor prognosis in melanoma patients [6], underlining the clinical relevance of MITF. The MITF gene encodes nine isoforms, which differ in tissue specificity and transcriptional targets [3, 7]. Although the melanocyte‐specific M isoform has been extensively studied, characterization of the other isoforms is lacking.

In addition to its oncogenic function in melanoma, deregulation of MITF expression has important implications in various cancer types [8, 9] highlighting the clinical relevance of MITF in nonmelanocytic cell lineages. MITF expression significantly impacts survival and resistance to CDK4/6 inhibitors in breast cancer [10]. In nonsmall cell lung cancer, high MITF expression correlates with improved prognosis. In line with that, silencing MITF in mouse models promotes metastasis and tumorigenesis [9], suggesting MITF suppresses tumor formation in lung cancer. In contrast to the oncogenic role of MITF, the mechanism underlying the tumor suppressive function of MITF remains unclear.

Genome instability refers to a high frequency of genetic alterations and is one of the major hallmarks of cancer. To maintain genome stability, dividing cells must faithfully duplicate their genome in each cell cycle and ensure an equal distribution of chromosomes to daughter cells. Replication stress is a broad term, which covers a variety of stresses that interfere with DNA replication and is recognized as the major source of genome instability in cancer [11, 12]. Replication stress can cause the DNA replication machinery to halt, which increases the risk of replication fork collapse and DNA double strand break (DSB) formation [13, 14]. Failure to complete DNA replication in S‐phase increases the risk of cells entering mitosis with under‐replicated DNA (urDNA), where it can cause aberrant segregation of chromosomes and DNA breakage [15]. Previous studies have shown that MITF plays a role in maintaining genome stability by controlling DNA replication, DNA repair, and mitosis in the melanocyte lineage [16, 17, 18].

The Hippo pathway is known to play a role in tissue growth through regulation of cell proliferation and apoptosis [19, 20]. Since its discovery, it has been shown that the Hippo pathway mediates cell cycle arrest and cell death in response to various stresses, including replication‐ and mitotic stress [21, 22, 23]. Therefore, it is not surprising that aberrant regulation of Hippo pathway signaling has been associated with tumorigenesis and tumor progression [24, 25, 26, 27]. Although primarily recognized as a key kinase of the Hippo signaling pathway, *Large tumor suppressor kinase 2* (LATS2) also regulates stress signaling independent of the pathway. In this context, LATS2 promotes P53 DNA binding in response to replication stress which results in transcription of apoptosis‐promoting genes [21, 28, 29]. Furthermore, in response to stress, LATS2 has been shown to promote rapid P53 protein accumulation by translocating to the nucleus where it binds and inhibits *Mouse double minute 2 homolog* (MDM2), an E3 ubiquitin ligase responsible for P53 proteasomal degradation [21, 23, 28, 29].

In this study, we show that depletion of MITF in nonmelanocyte cell lines negatively affects cell proliferation and DNA replication, leading to genome instability. We also show P53 activation upon MITF knockdown, which results in cell cycle arrest and apoptosis. Furthermore, we show that the observed P53 activation is mediated through LATS2, thereby uncovering an unrecognized connection between MITF and LATS2, a kinase that plays a key role in the Hippo pathway. Overall, our findings suggest that MITF plays an important role in maintaining genome stability in nonmelanocytic cell lineages, which might be important for the tumor suppressive function of MITF.

## 2 Materials and methods

### 2.1 Reagents

The following reagents were used: Aphidicolin (Aph) [Abcam (Cambridge, UK), ab142400]; KaryoMAX™ Colcemid™ [Thermo (Waltham, Massachusetts, USA), 15212012]; ATM inhibitor KU55933 [Sigma Aldrich (St. Louis, Missouri, USA), 587871‐26‐9]; ATR inhibitor VE‐821 (Sigma Aldrich, 1232410‐49‐9); AuroraA inhibitor JB300 (Tocris Bioscience, 7837/5); Nocodazole (Sigma Aldrich, SML1665); Doxycycline (Sigma Aldrich, D9891‐1G); Doxorubicin (Abcam, ab120629); Etoposide (Sigma Aldrich, E1383); and Karymax™ colcemid (Thermo, 15212012).

Following antibodies were used in this study: anti‐MITF C5 (Abcam, ab12039), anti‐53BP1 [Santa cruz (Dallas, Texas, USA), sc‐22760], anti‐γtubulin (Abcam, ab11316), anti‐p53 [Cell Signaling (Danvers, Massachusetts, USA), 2527], anti‐SMC1 (Abcam, ab9262), anti‐γH2AX (Abcam, ab22551), anti‐MYCtag (Cell Signaling, 2272), anti‐CyclinA (Santa Cruz, sc‐271 682), anti‐LATS2 (Abcam, ab243657), HRP 2° antibodies (Santa cruz) [anti‐mouse (sc‐2096), anti‐rabbit (sc‐2313)], IRDye 680LT anti‐mouse [Li‐Cor (Lincoln, NE, USA), 925‐6820], IRDye 800CW anti‐rabbit (Li‐Cor, 925‐32 211), Alexa Fluor 488 anti‐mouse 2° antibody [Life technologies (Carlsbad, California, USA), A21121], Alexa Fluor 488 anti‐rabbit 2° antibody [Invitrogen (Waltham, Massachusetts, USA), A32731], Alexa Fluor 555 anti‐rabbit 2° antibody (Invitrogen, A21428), Alexa Fluor 555 anti‐mouse 2° antibody (Invitrogen, A21422), Alexa Fluor 647 anti‐mouse 2° antibody (Invitrogen, A21235).

The following plasmid was used in this study: pClneoMYC‐LATS2 (66852) (Addgene).

The following kits and other reagents were used in the study: Lipofectamine^®^ RNAiMAX (Thermo Fisher, 13778075), Lipofectamine^®^ LTX with PLUS™ reagent (Thermo Fisher, 15338100), Pierce™ 16% Formaldehyde (W/V), methanol free (Thermo Fisher, 28906), Click‐It™ EdU kit (Thermo, C10419), RNeasy Mini kit (Qiagen, 74 104), TRI reagent (Thermo, AM9738), Superscript II (Thermo, 18 064022), SYBR green^®^ (Thermo, 25742), CellTrace™ Violet cell Proliferation Kit (Invitrogen, C34571), and caspase‐3/7 red dye [Sartorius (Göttingen, Germany), 4704].

The following specialized commercial instruments were used in the study: Olympus FV 1200 Confocal microscope, Attune NxT Flow cytometer (Thermo Fisher), Navios Ex (Beckman Coulter), CCD camera (Bio‐Rad ChemiDoc™ XRS+), Incucyte^®^ S3 live‐cell imager, and CFX Real‐time PCR Detection system (Bio‐Rad, Hercules, California, USA).

### 2.2 Biological resources

The following cell lines were used in this study: U2OS (human osteosarcoma, RRID:CVCL_0042), 624mel (human melanoma, RRID:CVCL_8054), Sk‐Mel‐28 (human melanoma, RRID:CVCL_0526), HeLa (human cervical cancer, RRID:CVCL_0030), A549 (human lung adenocarcinoma, RRID:CVCL_0023), D492 (human breast epithelia, RRID:CVCL_D1HJ), and SW1353 (chondrosarcoma, RRID:CVCL_0543).

### 2.3 Cell culture

Sk‐Mel‐28, 624mel, and derived cell lines were cultured in RPMI 1640 medium. U2OS, HeLa, SW1353, and A549 cells were cultured in Dulbebecco's modified Eagle medium [DMEM (1×) + Glutamax™ ‐I medium (Thermo, 31966047)]. All cancer cells were cultured in medium containing 10% fetal bovine serum (FBS) (Thermo, A5256801) and antibiotics [Penicillin 2 units/500 mL and Streptomycin 2 μg/500 mL (Thermo, 15070–063)]. The noncancer cell line D492 was cultured in a H14 medium [DMEM/F‐12 media (Thermo, 31330038)], 250 ng·mL^−1^ insulin (Sigma, 16634), 10 μg·mL^−1^ transferrin (Sigma, T8158), 10 ng·mL^−1^ EGF (PeproTech, 100–15), 2.6 ng·mL^−1^ sodium selenite (Sigma, S5261), 0.1 nM estradiol (Sigma, E2758), 0.5 μg·mL^−1^ hydrocortisone (Sigma, H0888) and 5 μg·mL^−1^ prolactin [PeproTech (London, UK), 100–07]. Cells were cultured in a humidified incubator at 37 °C with 5% CO_2_. All cell lines used in this study were authenticated within the past 3 years by STR profiling (Eurofins Genomics; 10 February 2026). U2OS, SK‐MEL‐28, HeLa, A549, and SW1353 cells were obtained from the American Type Culture Collection (ATCC). The 624mel cells were obtained from Dr. Steingrimsson, University of Iceland. D492 cells were obtained from (Dr. Gudjonsson, University of Iceland).

Genomic DNA from cell pellets was analyzed across 16 STR loci using PCR (AmpFlSTR^®^ Identifiler^®^ Plus kit), and profiles were matched to DSMZ and Cellosaurus databases according to ANSI/ATCC standards, with appropriate controls included. All lines showed high concordance with reference profiles, confirming identity. All experiments were conducted using mycoplasma‐free cells.

### 2.4 siRNA and plasmid transfection

Lipofectamine^®^ RNAiMAX (Thermo Fisher) was used for siRNA transfection according to manufacturer's recommendations. Briefly, transfection reagent was removed 24 h after transfection and fresh media added. The following siRNAs (Thermo Fisher, Silencer^®^ and Silencer select™) were used in this study: siMITF [s8791 (#1), s8792(#2)), siP53 (s606), siLATS2 (s25503 (#1), s25505 (#2)], siBRCA1(s458), siBRCA2 (s2085), si53BP1 (107785), siBLM (s1997) and siCON No. 1.

Lipofectamine^®^ LTX with PLUS™ reagent (Thermo Fisher) was used for plasmid transfections according to the manufacturer's recommendations.

### 2.5 Fluorescent microscope imaging

For immunofluorescence (IF) staining, cells were grown on autoclaved glass cover slips. Cells were fixed using 4% formaldehyde [diluted Pierce™ 16% Formaldehyde (W/V), methanol free (Thermo Fisher, 28 906)], for 15 min at RT. Cells were permeabilized in 0.2% Triton‐X [Merck (Rahway, New Jersey, USA), 108643] in phosphate‐buffered saline (PBS) for 4 min at RT and blocked in blocking buffer [DMEM (1×) + Glutamax™ ‐I medium + 10% FBS] for 30 min. Samples were incubated in primary antibody diluted in blocking buffer for 90 min at RT. Secondary antibody and 4′,6‐diamidino‐2‐phenylindole (DAPI) (Sigma, D9542) (1 μg·mL^−1^) staining was performed at RT for 60 min. Samples were washed in distilled water following two PBS washes and then dried. Coverslips were placed on a drop (4 μL) of mounting medium (Santa Cruz, sc516212) on a microscope slide. Click‐It™ EdU kit (Thermo, C10419) was used to mark cells that were actively replicating DNA, according to the manufacturer's guidelines. Olympus FV 1200 Confocal microscope was used for imaging. Automated image analysis was performed using the CellProfiler™ software. Protein intensity was measured using the mean intensity output of the MeasureObjectIntensity module. Nuclear and foci quantification was performed using the IdentifyPrimaryObject module. For image acquisition the 405 nm laser was used to identify DAPI stained nuclei, for visualization of the targets, fluorescent secondary antibodies were used (Alexa Fluor 488/555/647) in combination with image acquisition with lasers emitting light at 473 nm, 543 nm and 635 nm.

### 2.6 Flow cytometry

DNA content was stained with 7‐aminoactinomycin D (7AAD) (Thermo, A1310) or DAPI for flow cytometry analyses. Before staining cells were fixed using 70% ethanol. 1 × 10^6^ cells were resuspended in 500 μL of ice‐cold PBS, 1.2 mL ice‐cold ethanol was added drop wise while vortexing, and samples were then incubated for at least 3 h at −20 °C to complete fixation. After fixation samples were washed in PBS and resuspended in 300 μL of FACS staining buffer (0.1% Triton‐X in 1 × PBS) with 7AAD [25 μg·mL^−1^ (for 5 × 10^5^ cells)] or DAPI [5 μg·mL^−1^ (for 5 × 10^5^ cells)]. Samples were incubated in the dark for 30 min at RT. Samples were analyzed using the Attune NxT Flow cytometer (Thermo Fisher), Navios Ex [Beckman Coulter (Brea, California, USA)], and Flowjo™ software. Dye intensity was measured using Attune Flow cytometer (for DAPI: Ex. Laser: 405, Em. Filter: 440/50. for 7AAD: Ex. Laser: 561, Em. Filter: 620/15) (Thermo Fisher) (Ex. laser: 405, Em. filter: 440/50) and Flowjo (Thermo Fisher) software.

### 2.7 RNA sequencing

RNA was isolated using TRI reagent (Thermo, AM9738). The RNA was DNase1 treated [Qiagen (Hilden, Germany)] and purified using RNeasy Mini kit (Qiagen). The RNA samples were analyzed using Bioanalyzer [Agilent Technologies (Santa Clara, California, USA)]; all samples were found to have RNA integrity of 9.8 and higher. Paired and library sequencing was carried out on the Illumina platform. For gene expression quantification, we first cleaned original FASTQ files using Trimmomatic version 0.39 using default parameters [doi: 10.1093/bioinformatics/btu170] [30]. Next, we quantified gene expression from generated FASTQ files using kallisto version 0.50.1 [doi: 10.1038/nbt.3519] and the Ensembl Homo sapiens reference transcriptome (version 113). For differential gene expression analysis, first, we filtered out genes that were expressed at very low levels (max expression <1 TPM) and less than 20 reads in expression difference across conditions. Then, we used DESeq2 version 1.26.0 [doi: 10.1186/s13059‐014‐0550‐8] to determine statistically significant differentially expressed genes (DEGs) across experimental conditions (Benjamini–Hochberg correction *α* = 0.05; adjusted *P* < 0.05; |log_2_ FC| < 1). For functional enrichment analysis, we used clusterProfiler [doi: 10.1038/s41596‐024‐01020‐z] on Reactome Pathway ontology [doi: 10.1093/nar/gkad1025] to functionally characterize DEG sets. We accepted as enriched terms those with a *P* < 0.05 after multiple test correction. Code to reproduce all gene expression quantitative analysis is publicly available within GitHub repository https://github.com/adelomana/BMCBF/tree/main/research/025_isafjordur.

### 2.8 Metaphase spreads

Cells were cultured on 6‐well plates and harvested 7 days post siRNA treatment. To collect cells in metaphase, Colcemid was added to each sample 4 h before harvest. Pelleted cells were dissolved in a small volume of media; 2 mL hypotonic solution (0.075 M KCl) (37 °C) was then added dropwise to each sample while gently vortexing. Samples were incubated at 37 °C for 10 min. Cells were pelleted and dissolved in small volumes of the supernatant. Three milliliters of cold fixative (3:1 methanol and acetic acid) was added dropwise to each sample while vortexing; samples were then incubated on ice for 10 min. The fixative steps were repeated two to three times until the supernatant became clear. The pellets were resuspended in a small volume of fixative.

### 2.9 Western blotting

Whole cell lysates from cell culture (grown on 6‐well plates) were prepared by adding 100 μL of 2× Laemmli Sample Buffer (Santa Cruz, sc286963) to each sample and the cells scraped and moved to an Eppendorf tube. Benzonase (1 ng·mL^−1^) (Sigma, E1015‐5KU) was added before samples were heated at 95 °C for 7–8 min. Samples were blotted onto Nitrocellulose membrane (0.4 μm) (Santa Cruz, sc3724). Primary antibody incubation was performed O/N at 4° in PBS with 5% milk. Secondary antibodies were incubated for 60 min at RT in PBS with 5% milk. Membranes were treated with luminol reagent (Santa Cruz, sc2048) for 60 s, and images acquired using CCD camera (Bio‐Rad ChemiDoc™ XRS+).

### 2.10 qPCR

RNA was isolated using TRI reagent (Thermo, AM9738) and NanoDrop one (Thermo Scientific) was used to assess RNA concentration and purity. RNA samples were reverse transcribed using Superscript II (Thermo, 18064022) according to the manufacturer's protocol, using 1 ng isolated RNA and oligo (dT) in a total reaction volume of 19 μL, followed by RNase treatment [NEB (Ipswich, Massachusetts, USA), M0314L]. SYBR green^®^ (Thermo, 25742) amplification detection was used for the qPCR, and *β*‐actin was used to normalize data. All experiments were performed in technical triplicates and at least three biological replicates. Primer sequences are available in Data S2. CFX Real‐time PCR Detection system (Bio‐Rad) was used for qPCR experiments. For fold change calculations, the ΔΔCT method was used.

### 2.11 Cell proliferation assays

To assess cell proliferation, the CellTrace™ Violet cell Proliferation kit (Invitrogen, C34571) was used. The manufacturer's protocol was used for staining cells. Dye intensity was compared between treatments, where higher intensity indicated fewer cell divisions. Dye intensity was measured using the Attune Flow Cytometer (Thermo Fisher) (Ex. laser: 405, Em. filter: 440/50) and the FlowJo (Thermo Fisher) software.

### 2.12 Apoptosis assays

To analyze apoptosis, we used Incucyte^®^ S3 live‐cell imager (Sartorius) and caspase‐3/7 red dye (Sartorius, 4704). Cells were reverse transfected in 96‐well plates (3000 cells/well). After 24‐h siRNA treatment, the caspase‐3/7 dye (0.5 μm) was added. Cell confluency was obtained using the Incucyte^®^ S3 software, and Caspase‐3/7 positive cells were counted manually.

### 2.13 Survival assays

For survival and drug sensitivity assays, we used the Incucyte^®^ S3 live‐cell imager where cell confluency was analyzed using the Incucyte^®^ S3 software after 7 days of siRNA treatment.

### 2.14 Invasion assay

Eight‐micrometer pore transwell filters were used for the invasion assays [Corning (Corning, New York, USA)]. Twenty‐four hours after siRNA transfection, 40.000 cells were seeded on Matrigel (3% in serum free media) (Corning, 354230). Cells were given 20 h to invade through the Matrigel toward media containing 10% FBS. Invading cells were fixed in 4% formaldehyde for 5 min at RT and permeabilized in 0.1% Triton‐X for 5 min at 4 °C. Cells were stained in DAPI (1 μg·mL^−1^) diluted in PBS for 30 min at RT in the dark. Images were acquired using EVOS FL fluorescent microscope (Thermo Fisher Scientific).

### 2.15 CRISPR/Cas9 cell line

Custom recombinant lentiviral vectors were constructed and packaged by VectorBuilder. To generate the MITF knockout, a dual gRNA CRISP/Cas9 lentiviral vector was used (vector ID: VB260112‐1505guy (pLV[2CRISPR]‐hCas9:T2A:Puro‐U6>hMITF[gRNA20560]‐macU6>hMITF[gRNA20580])). For nontargeting control, a control lentiviral vector was used (vector ID: VB010000‐9492agg (pLV[Exp]‐EGFP/Puro‐EF1A>mCherry)).

U2OS cells were transfected with lentivirus at a multiplicity of infection (MOI) of 0.1 in media containing 5–10 μg·mL^−1^ polybrene. To select for cells with stable integration, puromycin was added 48 h (2 μg·mL^−1^) post‐transfection and maintained for an additional 48 h to ensure complete cell death of un‐transfected cells. Cells were grown with puromycin (0.5 μg·mL^−1^) after selection to prevent growth of potentially un‐transfected cells. MITF knockout was validated using qPCR.

### 2.16 Programs, software, algorithms and databases used in the study

Softwares and programs used in this study are as follows: CellProfiler cell image analysis software (https://cellprofiler.org/), FlowJo (v10) flow cytometry analysis software, Kaluza analysis 2.2 flow cytometry analysis software, Fiji image processing software (https://fiji.sc/). Following website was used in this manuscript: The Human Protein Atlas (HPA) (www.proteinatlas.org;https://www.proteinatlas.org/ENSG00000187098MITF/subcellular).

### 2.17 Statistical analysis

To compare the means of two groups, unpaired two‐tailed Student's *t*‐test was used to determine statistical significance. To compare the means of more than two groups, one‐way ANOVA was used. Error bars represented as the standard deviation. At least three biological replicates were used for each statistical analysis. Throughout the paper, statistical significance was presented as follows: *P < 0.05, **P < 0.01, ***P < 0.001, ***P < 0.0001.

## 3 Results

### 3.1 MITF promotes genome stability in nonmelanocyte cell lines

The role of the melanocyte‐specific MITF‐M isoform in melanocyte development and melanoma has been extensively studied. However, several other MITF isoforms have been found to be ubiquitously expressed in various tissue types, although their functional role remains to be characterized. Here, we hypothesized that MITF's role as a genome maintenance factor is not restricted to the melanocyte lineage. Firstly, we used information from The HPA to determine the mRNA expression of MITF across tissue types. According to the database, MITF is expressed in cell lines originating from several different tissues (Fig. 1A). For subsequent experiments, we selected four nonmelanocyte cell lines [HeLa (cervical cancer), U2OS (osteosarcoma), SW‐1353 (chondrosarcoma), and A549 (lung adenocarcinoma)] which according to HPA have MITF expression greater than 10 nTPM (normalized transcripts per million). To confirm specific MITF mRNA expression in the selected cell lines, we performed siRNA‐mediated MITF knockdowns. The results confirmed MITF mRNA expression in all cell lines tested (Fig. 1B). To further validate the nonmelanoma cancer cell line selection, the expression analysis was extended using the Cancer Cell Line Encyclopedia (CCLE, Ghandi et al. 2019, DepMAP 26Q1). The MITF expression levels of the cancer cell lines are consistent with the patterns provided by HPA (Fig. S1A). Abnormal gene expression is commonly seen in cancer cell lines, and therefore, MITF expression was also measured in the noncancer breast epithelia cell line, D492 [31] (Fig. 1B). Next, we compared the MITF mRNA expression values in the selected nonmelanocyte cell lines to the melanoma cell line (624mel). As expected, and in agreement with the HPA data, this revealed low but detectable expression of MITF in the nonmelanocyte cell lines compared with the melanoma cell line (Fig. 1C). Collectively, these data confirm that MITF is expressed in the nonmelanocyte cell lines tested, in low levels compared with the melanoma cell line 624mel.

**Fig. 1:**
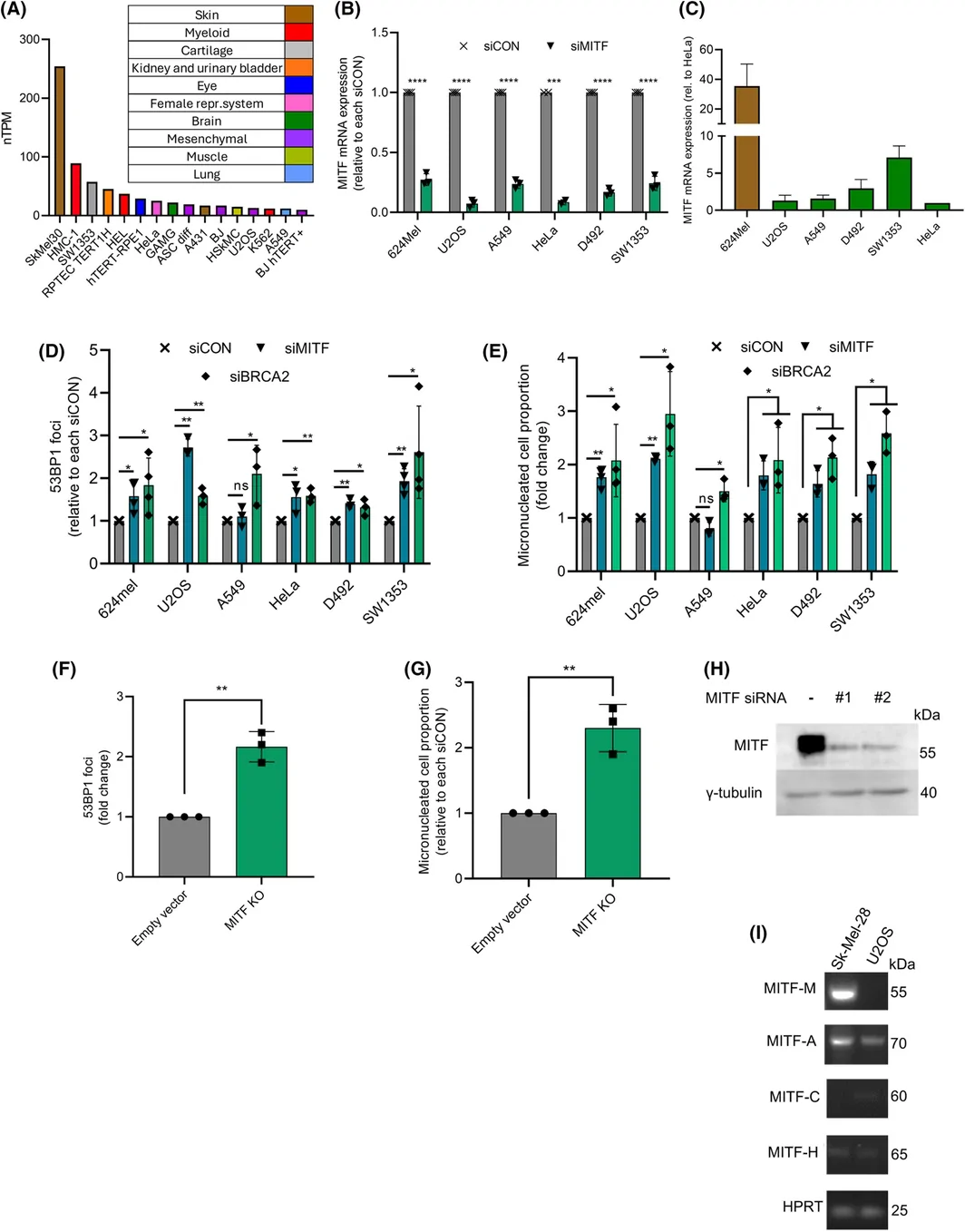
Microphthalmia‐associated transcription factor (MITF) knockdown causes genome instability in nonmelanocyte cell lines. (A) MITF normalized transcripts per million (nTPM) in cell lines originated from the indicated tissue types, data obtained from The Human Protein Atlas. (B) MITF mRNA expression confirmed in RNA samples extracted from four nonmelanocyte cancer cell lines (U2OS, A549, HeLa, and SW1353), a noncancer breast epithelia cell line (D492), and one melanoma cell line (624mel). Cells were treated with the indicated siRNAs for 48 h followed by real‐time qPCR analysis (unpaired *t*‐test, *n* = 3). (C) Comparison of mRNA expression levels in a melanoma cell line and nonmelanocyte cell lines using real‐time qPCR analysis. Expression values are shown as fold change, relative to HeLa expression values (*n* = 2). (D) Quantification of nuclear 53BP1 foci in the same cell lines as in b, following treatment with the indicated siRNAs for 72 h and immunostaining with 53BP1 specific antibody. The bar chart shows the fold change in 53BP1 foci formation compared with control cells for all five cell lines (unpaired *t*‐test, *n* = 3–4). (E) Analysis of the proportion of cells with micronuclei in the same cell lines as in (B), following 72‐h treatment with the indicated siRNAs and DAPI staining to visualize nuclear cells. The bar plot shows fold change in the proportion of cells with micronuclei compared with control cells for each cell line (unpaired *t*‐test, *n* = 3–4). (F) Quantification of nuclear 53BP1 foci in a CRISPR/Cas9 MITF knockout U2OS cell line and an empty vector control cell line. (unpaired *t*‐test, *n* = 3). (G) Analysis of the proportion of cells with micronuclei in a CRISPR/Cas9 MITF knockout U2OS cell line and an empty vector control cell line. (unpaired *t*‐test, *n* = 3). (H) MITF protein expression in U2OS cells treated with control and two independent MITF siRNAs, analyzed by western blot of whole cell extracts using the indicated antibodies. (I) Semi quantitative PCR bands obtained from MITF isoform‐specific primers. Data presented as mean ± SD. *P < 0.05, **P < 0.01, ***P < 0.001, ****P < 0.0001.

To determine the impact of MITF expression on genome stability in melanoma and nonmelanoma cell lines, the number of 53BP1 foci (marker for DNA DSBs) [32, 33] and the proportion of cells with micronuclei (marker for chromosome instability) [34, 35] after MITF knockdown was analyzed. MITF depletion resulted in a significant increase in 53BP1 foci and micronucleated cell counts in all cell lines tested with the exception of A549 (Figs 1D,E and S1B–C, S2, S3A). This strongly suggests that MITF plays a role in maintaining genome stability, which extends beyond the melanocyte lineage.

### 3.2 MITF supports DNA repair and cell cycle regulation in a nonmelanocyte cell line

Considering the strong indications of cellular stress detected in MITF‐depleted U2OS cells, we were interested in further estimating the degree of genome instability in this particular cell line. To validate the specificity of the observed phenotype, we generated a CRISPR/Cas9 MITF knockout U2OS cell line. The CRISPR‐mediated knockout recapitulated the results from the siRNA‐mediated MITF knockdown (Figs 1F,G and S3B–D). We confirmed MITF protein expression in U2OS cells using western blot analysis (Fig. 1H). Isoform expression analysis revealed that the MITF‐A isoform, which is the longest MITF isoform, is predominantly expressed in U2OS, although low MITF‐H expression was also detected. As expected, the melanocyte‐specific M isoform was not detected in U2OS cells (Fig. 1I and Data S1).

To evaluate the impact of MITF on cell integrity, we performed metaphase spreads to measure the level of chromosomal instability in MITF‐depleted cells. The metaphase spreads revealed that MITF knockdown resulted in a significant increase in chromosome aberrations compared with control cells, which was a similar increase as was observed with BRCA2 knockdown or induction of mild replication stress by low dose DNA polymerase inhibitor treatment (aphidicolin 200 nm for 24 h) (Figs 2A and S4A).

**Fig. 2:**
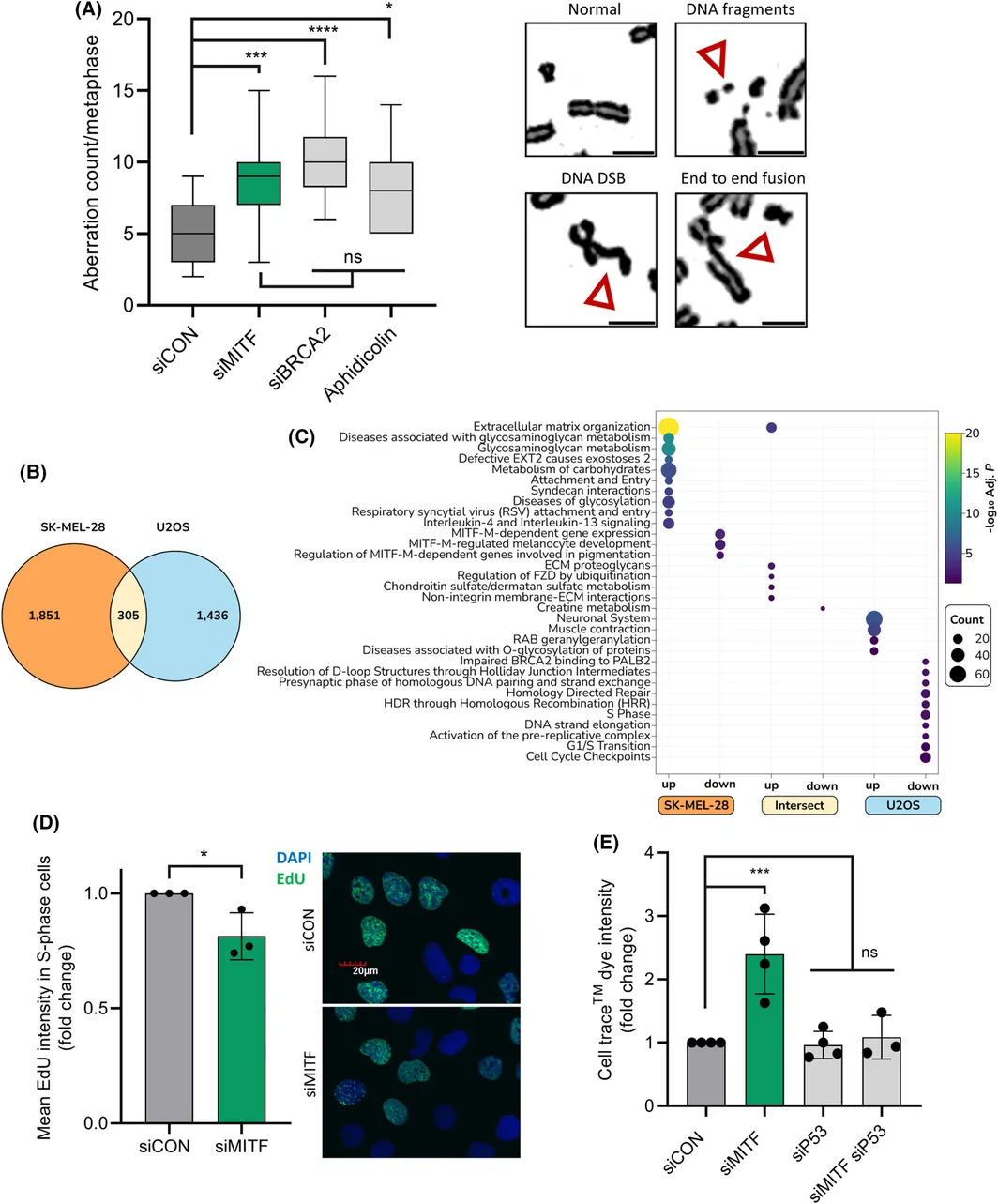
Microphthalmia‐associated transcription factor (MITF) knockdown has an impact on genome stability, DNA replication and cell proliferation in U2OS cells. (A) Quantification of chromosome aberrations (right panel shows examples of aberrations observed) in metaphase spreads samples after seven‐day siRNA (siCON, siMITF, and siBRCA2) treatment or 24‐h Aphidicolin treatment (200 nm). To enrich for cells in metaphase, samples were treated with the mitotic inhibitor Colcemid 4 h prior to fixation. Scale: 10 μm (Welch's test, *n* = 7–23). (B) Venn diagram comparing differentially expressed gene sets responding to MITF depletion in two different cell lines. (C) Dot plot showing a nonredundant summary of functionally enriched pathways for the different gene sets indicated. (D) Mean EdU intensity in S‐phase cells after 48‐h siRNA treatment (siCON and siMITF) and a 30 min incubation with EdU, followed by a click chemistry staining of EdU and DAPI staining to visualize nuclear cells. Scale: 20 μm (unpaired *t*‐test, *n* = 3). (E) Violet blue dye intensity after five‐day siRNA treatment (siCON, siMITF, siP53, and siMITF + siP53). Prior to siRNA transfection, cells were stained with a Violet blue cell trace™ dye. Dye intensity was obtained using flow cytometry (gating: single cells‐violet dye histogram), higher intensity indicating fewer cell cycles (one‐way ANOVA, *n* = 3). Data presented as mean ± SD. *P < 0.05, **P < 0.01, ***P < 0.001, ****P < 0.0001.

Therefore, we sought to characterize the molecular mechanisms behind the observed cellular response to MITF depletion in U2OS cells. Toward this goal, we compared the transcriptional response to MITF knockdown in U2OS with SK‐MEL‐28, which is a cell line of melanocytic origin. We identified (see Methods for details) a similar number of DEGs in both cell lines upon MITF loss: 2156 DEGs in SK‐MEL‐28 and 1741 DEGs in U2OS (Table S1, Fig. S4B). Yet, the intersection between these two sets is relatively small: Upon perturbation, only 305 DEGs (14% of DEGs in SK‐MEL‐28) are shared between the two cell lines (Fig. 2B). Thus, we hypothesized that the molecular mechanisms behind the two transcriptional responses may be different. To further characterize the perturbed functions in each cell context, we performed pathway enrichment analysis on the DEGs particular and common to each cell line (Table S2). We identified two clearly distinct responses (Fig. 2C). The response in SK‐MEL‐28 is characterized by the upregulation of genes involved in the remodeling of the extracellular matrix as supported by pathway terms, such as *Extracellular matrix organization* (*P* < 2 × 10^−21^), *Metabolism of carbohydrates* (*P* < 2 × 10^−6^), and *Syndecan interactions* (*P* < 2 × 10^−5^). The set of downregulated genes in SK‐MEL‐28 is enriched on MITF‐specific targets only, such as *MITF‐M‐dependent gene expression* (*P* < 5 × 10^−4^), *MITF‐M‐regulated melanocyte development* (*P* < 2 × 10^−3^), and *Regulation of MITF‐M‐dependent genes involved in pigmentation* (*P* < 5 × 10^−5^).

However, the transcriptional response in U2OS is unequivocally different. Most of the significantly enriched pathways in the downregulated DEG set (18 out of a total of 25 enriched pathways) elicit DNA repair functions, such as *Homology Directed Repair* (*P* < 8 × 10^−3^) and *Diseases of DNA repair* (*P* < 2 × 10^−2^). Six other significantly enriched pathways revolve around cell cycle progression, as supported by the terms *S‐Phase* (*P* < 8 × 10^−3^), *G1/S Transition* (*P* < 4 × 10^−2^), and *Cell Cycle Checkpoints* (*P* < 4 × 10^−2^) with key genes, such as *MCM4, ORC1, FEN1, RFC1, ORC*1, and *CDT1* as clear replication hallmarks. Altogether, these results indicate that the response to MITF loss in U2OS is dissimilar compared with a melanocytic cell background, and it is indeed markedly characterized in U2OS by the downregulation of genes involved in DNA repair and replication.

Next, we aimed to experimentally validate the impact of MITF on DNA repair and replication in the U2OS cell line. To determine the proportion of DNA replicating cells, U2OS cells were treated with a short pulse of the thymidine analog EdU, followed by image analysis to determine the rate of EdU incorporation. Interestingly, we detected a drop in EdU incorporation, specifically, a lower mean intensity of EdU in the S‐phase of MITF‐depleted cells compared with control cells, indicative of reduced DNA replication (Fig. 2D). Consistent with reduced EdU incorporation, we observed weaker Ki67 signal following MITF knockdown, further suggesting slower cell cycling (Fig. S4C). Additionally, it is well known that MITF knockdown affects melanoma cell proliferation [1, 2, 3, 4, 5]. Hence, we investigated whether MITF knockdown affects cell proliferation in U2OS cells. We used CellTrace™ Violet dye to trace cell proliferation in MITF‐depleted cells. We stained cells with the CellTrace™ dye prior to siRNA treatment that dilutes with each cell division. In this experiment, the intensity of the cell dye was approx. 2.5‐fold stronger in the MITF knockdown cells compared with a negative control, indicating a reduced number of cell divisions after MITF knockdown (Fig. 2E). Taken together, these results support that reduced MITF expression in U2OS cells causes chromosomal instability and negatively affects DNA replication and cell proliferation.

### 3.3 MITF expression promotes stable DNA replication

Halted DNA replication can cause replication stress and urDNA when cells enter mitosis, which can potentially lead to the formation of bulky anaphase bridges (BABs) [15]. As the cell divides, the enmeshed DNA can then give rise to DNA DSBs in the subsequent G1 phase; this damage forms structures known as 53BP1 nuclear bodies (NBs) [36, 37]. To assess the impact of MITF depletion on replication stress in U2OS cells, we used automated image analysis to quantify the number of 53BP1 foci in G1 phase (cyclin A negative cells). We found an increase in both BABs and 53BP1 NBs in MITF knockdown U2OS cells, indicative of increased replication stress after MITF loss (Fig. 3A,B). To exclude potential siRNA off‐target effects, we repeated the experiments in U2OS CRISPR KO cells as well as in 624mel cells expressing a dox‐inducible MITF micro‐RNA construct [38]. Both CRISPR mediated loss and inducible KD of MITF recapitulated the phenotype observed with siRNA‐mediated depletion in U2OS cells, further supporting a role for MITF in suppressing genomic instability in a nonmelanoma cell line (Figs 3C,D and S5A–D).

**Fig. 3:**
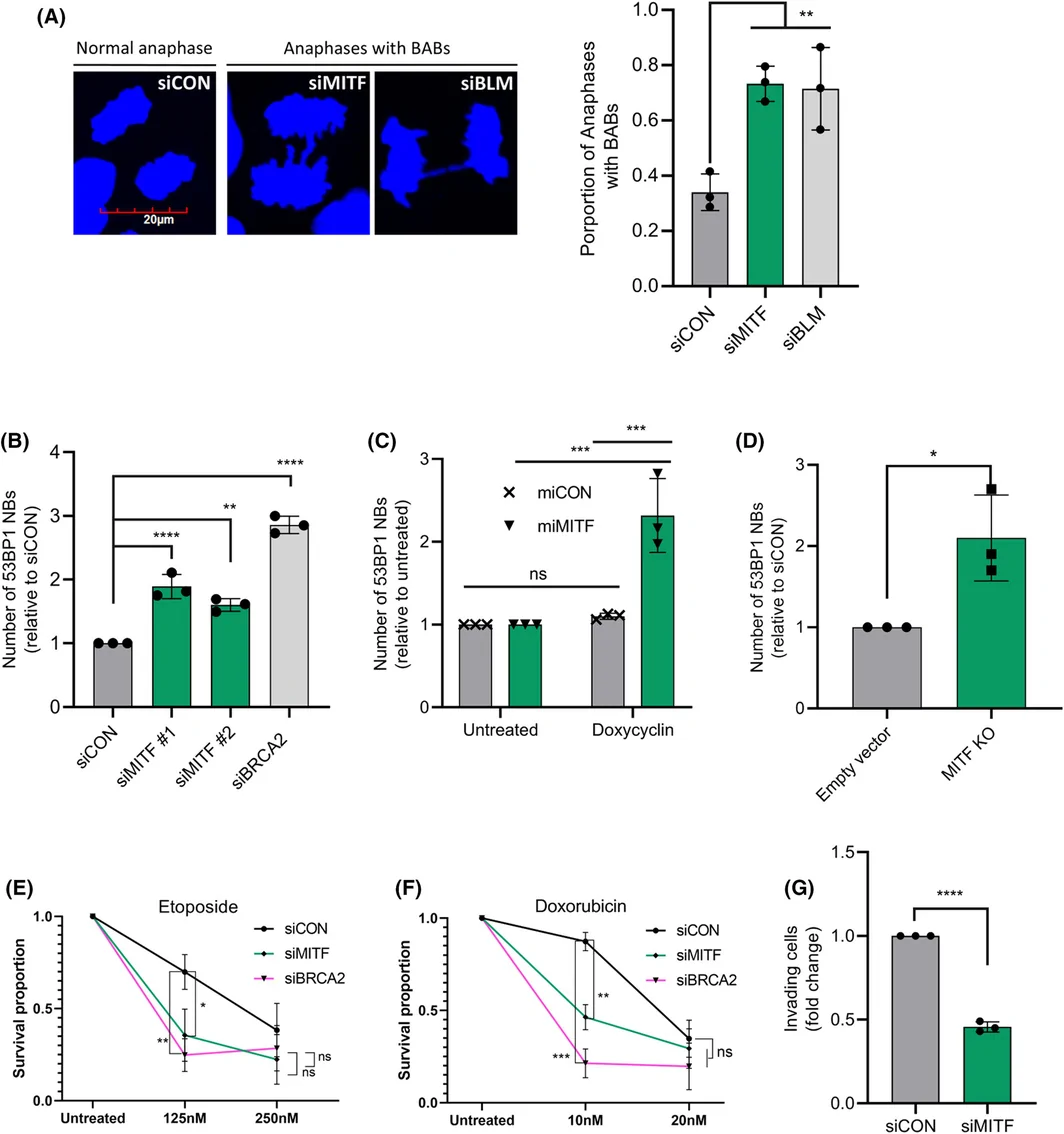
Microphthalmia‐associated transcription factor (MITF) knockdown in U2OS cells leads to replication stress and increased drug sensitivity. (A) Quantification (left) and representative images (right) of bulky anaphase bridges (BABs) after 72‐h siRNA treatment (siCON, siMITF, and siBLM) followed by DAPI staining. Bar plot shows proportion of anaphase cells with BABs. Scale: 20 μm (one‐way ANOVA, *n* = 3). (B) Quantification of 53BP1 nuclear bodies after 48‐h siRNA treatment (siCON, siMITF, siBRCA1, si53BP1), followed by immunostaining with antibodies targeting 53BP1 and CyclinA. Scale: 20 μm (one‐way ANOVA, *n* = 3). (C) Quantification of 53BP1 nuclear bodies after 48‐h Doxycycline inducible MITF knockdown in 624mel cells, followed by immunostaining with antibodies targeting 53BP1 and CyclinA. Scale: 20 μm (one‐way ANOVA, *n* = 3). (D) Quantification of 53BP1 nuclear bodies in a CRISPR/Cas9 MITF knockout U2OS cell line and an empty vector control cell line (unpaired *t*‐test, *n* = 3). (E) Survival assay after seven‐day siRNA treatment (siCON, siMITF and siBRCA2 as positive control) and Etoposide treatment. Cell survival was obtained with live‐cell imaging by measuring cell confluency. The graph shows proportion of surviving cells compared with untreated samples (unpaired *t*‐test, *n* = 4). (F) Survival assay after seven‐day siRNA treatment (siCON, siMITF, and siBRCA2) and Doxorubicin treatment. Survival data presented as in d (unpaired *t*‐test, *n* = 3). (G) Matrigel invasion assay after 48‐h siRNA treatment (siCON and siMITF). Number of invading cells were obtained with EVOS FL fluorescent microscope following DAPI staining (unpaired *t*‐test, *n* = 3). Data presented as mean ± SD. *P < 0.05, **P < 0.01, ***P < 0.001, ****P < 0.0001.

### 3.4 MITF‐depleted cells are hypersensitive to topoisomerase II inhibitors

Genome instability is in many cases associated with increased sensitivity to genotoxic agents. To test whether increased chemotherapeutic drug sensitivity was observed after MITF knockdown, we used live‐cell imaging to measure cell survival following drug treatment. We tested sensitivity to the topoisomerase II inhibitors doxorubicin and etoposide in U2OS cells, which are both drugs used in the treatment of various cancers [39]. We found that cell survival was lower after etoposide (Fig. 3E) and doxorubicin treatment (Fig. 3F) in MITF knockdown cells, indicating that MITF expression is important for cell survival in response to commonly used chemotherapeutic agents.

According to the MITF rheostat model, MITF is believed to play a role in the reversible switch between melanoma cell phenotypes. According to this model, low MITF expression is suggested to be associated with cell invasion [40]. To test whether MITF influenced the invasiveness of U2OS cells, we performed a Matrigel transwell invasion assay. Knockdown of MITF resulted in a clear reduction in the number of invading cells (Figs 3G and S5E).

Collectively, these data show that upon MITF knockdown, U2OS cells have decreased invasiveness and are more sensitive to chemotherapeutic drug treatment.

### 3.5 MITF knockdown activates the P53 stress signaling pathway

P53 plays a key role in cell cycle regulation and apoptosis in response to DNA damage [41]. Given the observed genome instability phenotypes, we decided to look at P53 activation before and after MITF depletion. Knockdown of MITF, using siRNAs, resulted in a twofold increase in P53 protein levels and a significant increase in mRNA expression (Fig. 4A,B). Consistent results were obtained in the melanoma cell line 624mel, following RNAi and doxycycline inducible knockdown of MITF (Fig. S6). Furthermore, MITF knockdown in U2OS cells affected cell cycle distribution, whereas an increased proportion of cells were observed in G1‐phase, indicating cell cycle arrest (Fig. 4C). Importantly, in the isogenic U2OS P53 knockout cell line [42] no G1 arrest is observed, supporting the notion that the cell cycle arrest is mediated by P53 (Fig. S7A).

**Fig. 4:**
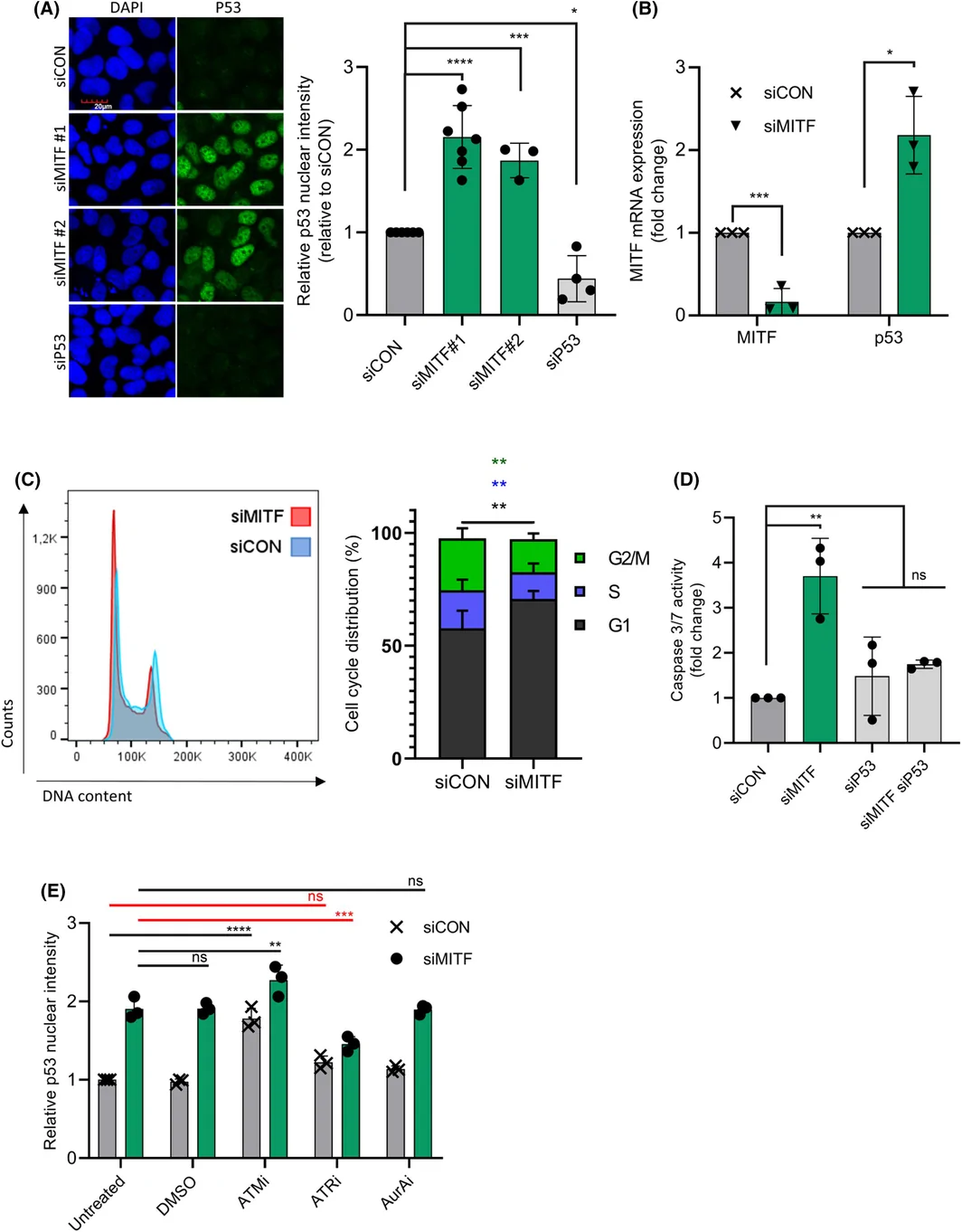
P53 is activated upon microphthalmia‐associated transcription factor (MITF) knockdown in U2OS cells. (A) Confocal microscopy images and quantification of P53 protein levels after 48‐h treatment with siCON, siMITF, and siP53, followed by immunostaining with antibody targeting P53 and DAPI to visualize nuclear cells. Scale: 20 μm (one‐way ANOVA, *n* = 3–7). (B) MITF and p53 mRNA expression in U2OS cells. Cells were treated with indicated siRNAs for 48 h, followed by real‐time qPCR analysis. (unpaired *t*‐test, *n* = 3). (C) Cell cycle profile analyses of siCON and siMITF treated U2OS cells. Cells were fixed after 48‐h siRNA treatment, followed by staining of DNA content with 7‐aminoactinomycin D (7AAD) and flow cytometry analysis (gating: Single cells‐7AAD) (unpaired *t*‐test, *n* = 4). (D) Caspase 3/7 activity relative to cell confluency after 4 days of siRNA treatment (siCON, siMITF, sip53, siMITF + sip53). Cells were incubated with caspase‐3/7 red dye after siRNA treatment and imaged in IncuCyte® live‐cell imager (one‐way ANOVA, *n* = 3). (E) quantification of P53 protein levels after 48 h siCON and siMITF treatment and 24‐h treatment with ATMi, ATRi, AuroraAi, or DMSO, followed by immunostaining with antibody targeting P53 and DAPI to visualize nuclear cells (one‐way ANOVA, *n* = 3). Data presented as mean ± SD. *P < 0.05, **P < 0.01, ***P < 0.001, ****P < 0.0001.

To verify whether MITF knockdown would affect the survival of the U2OS cell line, we next sought to evaluate whether the cells would enter an apoptotic state upon treatment with MITF siRNA. Caspase cleavage assay revealed an increase in apoptosis after MITF knockdown, with an approx. fourfold increase in caspase cleavage activity after 96 h siRNA treatment (Fig. 4D). Collectively, these data suggest that MITF‐depleted cells activate the P53‐mediated stress response, resulting in cell cycle arrest and apoptosis. These results are also in line with the cell proliferation analysis, whereas reduced cell proliferation of MITF‐depleted cells is rescued with a double knockdown of MITF and P53 (Fig. 2E).

To further characterize the upstream mechanism of the P53 stress response observed in MITF knockdown U2OS cells, we inhibited key members of the phosphoinositide‐3‐kinase‐related protein kinase (PIKK) family, which play a central role in the DNA damage response, as well as the mitotic kinase AuroraA, which is known to phosphorylate LATS2 in mitosis [43, 44]. Inhibiting ATM and AuroraA had no effect on P53 accumulation following MITF knockdown. However, inhibiting ATR, a key kinase activated during replication stress, significantly reduced P53 accumulation, suggesting that problems during replication are involved in P53 activation in MITF‐depleted cells (Fig. 4E).

Furthermore, given the observed connection between MITF knockdown and P53 expression, we used TCGA to determine the correlation in mRNA expression between these two genes in cancer. Out of 32 different cancer types, 18 showed a negative correlation between MITF and p53 (sarcoma shown as an example, Fig. S7B).

### 3.6 MITF knockdown promotes P53 activation and cell cycle arrest through LATS2

Upon cellular stress, P53 is activated by tightly regulated protein modifications, which rapidly results in P53 protein accumulation and subsequent cell cycle arrest. P53 is phosphorylated at various sites, causing conformational changes, which blocks ubiquitination‐mediated degradation by MDM2 [45, 46, 47]. It has also been shown that in response to mitotic stress and chromosomal instability, LATS2 is translocated to the nucleus where it binds MDM2 and inhibits its ligase activity toward P53, resulting in rapid P53 accumulation [21, 23, 28, 29]. As we show above, MITF knockdown yields a dramatic increase in P53 expression and increased number of chromosome aberrations (Figs 4A and 2A). Therefore, we investigated if P53 activation in MITF‐depleted cells is dependent on LATS2 activity. To this end, we did double knockdowns of MITF and LATS2 in U2OS cells to examine the impact on P53 expression and cell cycle arrest. We found that co‐depletion of MITF and LATS2 resulted in a complete rescue of both P53 protein accumulation and cell cycle arrest (Figs 5A–B and S8, S9A–B), suggesting strong dependency on LATS2. Additionally, co‐depletion of MITF and LATS2 in 624mel cells partially rescued the P53 protein accumulation (Fig. S9C). Attempting to rescue G_1_ cell cycle arrest in the 624mel cell line was not possible since the cells are not arrested in the G_1_ phase upon MITF depletion (Fig. S8C). This is most likely due to a mutation in the DNA binding domain of P53 in 624mel cells (https://www.cellosaurus.org/CVCL_8054). To confirm LATS2 activation, we analyzed nuclear translocation of Myc‐LATS2 in control and MITF knockdown cells. This revealed a threefold increase of LATS2 in the nucleus in MITF‐depleted cells, indicating LATS2 activation (Fig. 5C). qPCR analysis revealed a modest upregulation of LATS2 mRNA after MITF knockdown (Fig. 5D), suggesting MITF affects LATS2 on transcription level.

**Fig. 5:**
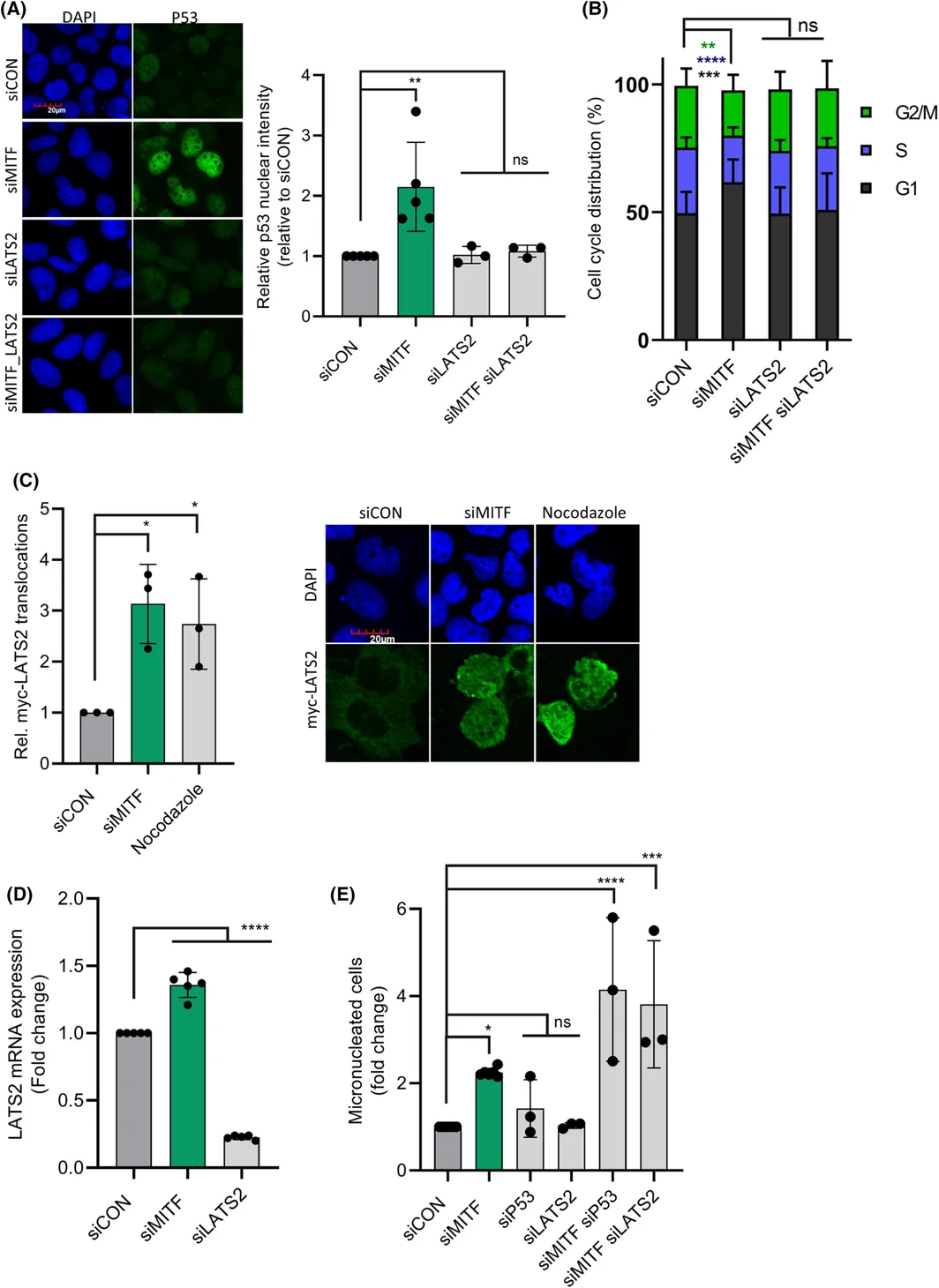
P53 activation in microphthalmia‐associated transcription factor (MITF) knockdown cells is dependent on the Hippo pathway kinase LATS2. (A) Confocal microscopy images (left) and quantification (right) of P53 protein levels in U2OS cells 48 h after treatment with siCON, siMITF, siLATS2, and siMITF + siLATS2, cells were fixed and immunostained with a P53 specific antibody and DAPI to visualize nuclear cells. Scale: 20 μm (one‐way ANOVA, *n* = 3–5). (B) Cell cycle profile analyses of siCON, siMITF, siLATS2, and siMITF + siLATS2 treated U2OS cells. Cells were fixed after 48‐h siRNA treatment, followed by staining of DNA content with 7‐aminoactinomycin D (7AAD) and flow cytometry analysis (gating: Single cells‐7AAD) histogram (one‐way ANOVA, *n* = 4). (C) Confocal images (right) and quantification (left) of Myc‐LATS2 translocation into the nucleus in siCON and siMITF treated U2OS cells. Twenty‐four‐hour Nocodazole treatment was used as a positive control. Cells were treated with siRNA for 48 h, Myc‐LATS2 plasmid transfection was performed 24 h prior to fixation. This was followed by immunostaining with Myc‐tag‐specific antibody and DAPI to visualize nuclear cells. Scale: 20 μm (one‐way ANOVA, *n* = 3). (D) MITF and LATS2 mRNA expression in U2OS cells after 48 h siCON and siMITF treatment. mRNA levels were analyzed using qPCR (one‐way ANOVA, *n* = 4). (E) Quantification of the proportion of cells containing micronuclei following 72 h siRNA treatment (siCON, siMITF, siP53, siLATS2, siMITF + siP53, and siMITF + siLATS2) and DAPI staining. The bar plot shows fold change in cells with micronuclei compared with siCON (one‐way ANOVA, *n* = 3). Data presented as mean ± SD. *P < 0.05, **P < 0.01, ***P < 0.001, ****P < 0.0001.

As a key activator of DNA damage checkpoint signaling, P53 plays an important role in preventing replication in damaged cells and subsequent loss of genetic integrity. Accordingly, we observed increased genome instability after MITF knockdown, when P53 activation was inhibited by co‐depleting the cells with siLATS2 or siP53 (Fig. 5E). Furthermore, we found that LATS2 or P53 knockdown alone had no effect on genome instability; it only affects the cells in combination with MITF depletion. This supports that LATS2 dependent activation of P53 plays a key role in counteracting genome instability caused by reduced MITF expression.

### 3.7 Low MITF expression is associated with poor survival in nonmelanoma cancer

MITF has a well‐established role as an oncogene in melanoma. Indeed, increased MITF expression has been associated with poor prognosis in melanoma patients [6]. Therefore, we sought to investigate the effect of MITF expression on patient survival in nonmelanoma cancers. Using data from the Cancer Genome Atlas (TCGA), we generated Kaplan–Meier plots in patient cohorts separated by MITF gene expression levels. MITF expression is associated with survival in sarcoma, adrenocortical carcinoma, and kidney clear cell carcinoma as well as melanoma (Fig. S10). This shows that changes in MITF expression are clinically relevant in melanoma and nonmelanoma cancers. Conclusively, low MITF expression is associated with poor survival in the nonmelanoma cancer types, which may be indicative of a tumor suppressive role of MITF.

## 4 Discussion

Despite a well‐illustrated role of MITF in melanocytes and melanoma, it has been shown that MITF has a role in osteoclast and mast cell development, olfactory bulb function, autophagy, and retinal pigment epithelium (RPE) development [7, 48, 49, 50, 51, 52, 53, 54]. In recent years, MITF has also been suggested to have melanocyte‐specific roles in mitotic regulation, DNA replication, and DNA damage repair, all of which are important to maintain genome integrity [16, 17, 18]. Here, we show that MITF has an important function in maintaining genome stability in cell lines derived from osteosarcoma (U2OS), chondrosarcoma (SW‐1353), cervical cancer (HeLa), and the noncancer breast epithelia cell line D492. This suggests that MITF preserves genome integrity in a variety of tissue types and is not limited to cells of melanocytic origin. We show that nonmelanocyte cell lines that express low levels of MITF compared with melanoma cell lines depend on MITF to maintain their genome integrity (Figs 1D–G and S1–S3). It should, however, be noted that MITF quantification by qPCR relied on single‐gene ΔΔCt normalization, which, while widely used, does not conform to MIQE guidelines recommending the use of multiple validated reference genes.

Genome instability is considered one of the hallmarks of cancer and a major driving force for tumorigenesis. In light of that, our results indicate that in addition to its established role as a melanoma specific oncogene, MITF might also play a broader role as a genome maintenance factor, which can potentially contribute to the recently described tumor suppressive function of MITF [55].

Although our results clearly show that MITF is important for maintaining genome stability in various tissue types, there might be examples where other mechanisms are at play. One out of the six cell lines tested, A549, did not show any signs of genome instability following MITF knockdown (Fig. 1D,E). Mechanistically, we do not understand the lack of phenotype in A549. Potentially, A549 harbors a mutation or an epigenetic modification which neutralizes the damaging effect of MITF depletion. Nevertheless, these results are interesting since they show that there is a level of tissue specificity involved in MITF's function in genome maintenance, which merits further research.

We also show that MITF knockdown negatively affects cell proliferation of U2OS cells and that long‐term MITF depletion ultimately results in apoptosis (Figs 2E and 4D). Our findings show an impairment in cell cycle progression, with less cells in S‐phase and slower incorporation of nucleotides upon MITF knockdown in U2OS cells (Fig. 2C,D 4C, and Tables S1 and S2), indicating decreased DNA replication. Markedly, it has been reported that DNA replication is under MITF regulation in melanoma cells [18]. Moreover, our data suggest that the stress response observed in MITF knockdown cells is associated with ATR and LATS2 (Figs 4E, 5A–E and S5A–G), revealing a novel connection between MITF and key signaling factors in the response to DNA damage. From a mechanistic perspective, the possible connection with ATR gives important insight into the source of genome instability in MITF deficient cells. The ATR kinase is activated in response to replication‐associated stress and plays a key role in maintaining the integrity of DNA replication under stress conditions [11, 13]. The requirement for ATR in LATS2/P53 associated stress signaling is consistent with a contribution of replication‐associated stress to genome instability in MITF deficient cells.

Our data suggest a relationship between ATR activity and nuclear p53 accumulation in MITF‐depleted U2OS cells, but the underlying mechanism remains unresolved. While ATR is a central regulator of cellular stress responses during DNA replication, we do not directly demonstrate that the chromosomal instability phenotypes observed here, including anaphase bridges and 53BP1 nuclear bodies, are ATR‐dependent. Therefore, alternative sources of genome instability cannot be excluded. In particular, defects in sister chromatid cohesion or mitotic progression, which are known to induce a strong LATS2‐dependent P53 response, could also contribute to the formation of anaphase bridges and unresolved DNA lesions [23, 56].

Furthermore, p53 activation downstream of ATR may reflect indirect effects on cell‐cycle progression or broader stress signaling pathways rather than a direct mechanistic link. Therefore, while our data support the involvement of ATR signaling in the stress response observed upon MITF depletion, they do not establish a causal pathway connecting ATR activity to the observed chromosomal instability phenotypes.

Future studies incorporating ATR inhibition in combination with direct readouts of chromosomal instability will be required to resolve these mechanisms.

Previously, it has been shown that MITF directly regulates genes required for DNA replication in melanoma cells [17, 18, 57]. This is in accordance with our data, where DNA replication pathways scored as significantly enriched terms in the set of downregulated genes in U2OS MITF knockdown cells. Interestingly, major DNA repair pathways were also significantly enriched, pointing to a potential mechanism for impaired cell replication (Fig. 2C and Table S2).

As expected, our data revealed expression of both the A‐ and the M isoform in the melanocytic Sk‐Mel‐28 cell line. Interestingly, the U2OS cell line expresses the A‐ and the H‐isoforms, whereas the A isoform is predominantly expressed (Fig. 1I). This raises the question of whether different MITF isoforms carry out different functions. The majority of studies on MITF in melanoma or melanocytes do not differentiate between isoforms. However, in a recent study, an isoform‐specific function of the A isoform as a transcriptional regulator of genes involved in autophagy and oxidative metabolism was observed [58]. Furthermore, a study using isoform‐specific knockout mice reveals that the M isoform specifically contributes to pigmentation, while the knockout of the A isoform had only a minor effect on that phenotype [59, 60]. The phenotype reported here is most likely designated to the A isoform as it is the MITF isoform predominantly expressed in the U2OS cell line; however, this does not exclude the possibility of other MITF isoforms participating in genome stability.

Melanoma is an aggressive cancer type with high metastatic potential and poor 5‐year survival rates [6]. MITF is proposed to act as a rheostat by controlling the switch between different signaling pathways that determine the phenotype of melanoma cells [40] making melanoma frequently resistant to therapeutic treatments [4, 5, 61]. According to the rheostat model, high MITF levels in melanoma cells are linked to a proliferating phenotype, while cells expressing lower levels of the protein are less proliferative and more invasive, and MITF depletion ultimately results in cell death. However, more recent studies have not been able to replicate the more invasive phenotype in *in vitro* studies [38, 62]. In these studies, MITF knockdown in melanoma cells has little to no effect on invasion ability, but knockout results in less invasion ability. Our data suggest a significant reduction in invasive potential (Fig. 3G), but like melanoma, osteosarcoma has high metastatic rates and 5‐year survival of metastatic patients as low as 20% [63, 64]. It should be noted that the tumor microenvironment plays an important role in tumor cell invasion [61], which our experimental settings are not able to simulate. Therefore, the reduced ability to invade through the Matrigel might be a consequence of the overall reduced fitness of the MITF knockdown cells.

Results from our cell line analysis (Fig. 1) indicate that MITF has an important function in melanoma and nonmelanoma cancers. In line with that, we show that MITF expression has a significant effect on survival in various nonmelanoma cancers (Fig. S10). This supports our hypothesis and underscores the importance of characterizing the role of MITF across cell lineage. Furthermore, results from our drug sensitivity experiments revealed that MITF knockdown increases sensitivity of U2OS cells to the chemotherapeutic drugs, Etoposide and Doxorubicin (Fig. 3E,F) [65], which indicates that MITF levels might determine treatment outcomes not only in melanoma patients but in other cancer patients as well.

As transcription factor, MITF regulates the expression of a large number of genes, with approximately 500 genes as validated targets [66]. Functionally, MITF target genes are quite diverse, including genes involved in pigmentation, DNA damage response, mitotic regulation and DNA replication [18, 66]. Our data suggest that the role of MITF as a regulator of genome maintenance extends beyond the melanocyte cell lineage by regulating expression of genes with roles in DNA replication and DNA repair (Fig. 2C, Tables S1 and S2). These transcriptional targets of MITF have been reported in melanoma cells on many occasions in the literature [16, 17, 18], but here, we propose that this function is not melanocyte‐specific, suggesting MITF expression is biologically relevant in other tissue types. Dysregulation of LATS2 expression and the Hippo pathway, has been linked to tumorigenesis and cancer progression in many cancer types, [24, 25, 26, 27, 67, 68, 69, 70]. Here, we show a novel connection between MITF and LATS2, a key kinase in the Hippo pathway, which in light of its important role in tumor progression, further supports the potential tumor suppressive role of MITF.

## Conflict of interest

The authors declare no conflicts of interest.

## Author contributions

TG and StS were responsible for study design. DHG contributed to the study design, performed confocal imaging assays, flow cytometry assays, Matrigel trans well assays, and live‐cell imaging assays along with manuscript writing. TG, StS, LV, and ALGM contributed to manuscript writing. RNA sequencing and analysis was performed by SnS, KK, ALGM, and DM. Isoform analysis was performed by KK. ESv and SR performed the metaphase spreads and aided in the analysis of chromosome images. Doxycycline inducible cell lines were designed and made by RD. CRISPR/Cas9 cell line was generated by TBV. TG and DHG performed WB analyses and TBV, DHG, and MRB performed qPCR analyses. Data interpretation was done by DHG, TG, StS, and ALGM. ESt contributed intellectually to the study and writing of the manuscript.

## Supporting information


**Table S1.** Differentially expressed genes following MITF depletion in Sk‐Mel‐28 cells.
Table S1 Differentially expressed genes following MITF depletion in U2OS cells.

**Table S2.** Reactome pathway analysis in Sk‐Mel‐28 and U2OS cells.

**Fig. S1.** MITF knockdown causes genome instability in nonmelanocyte cell lines. (A) MITF expression distribution across 1719 cancer cell lines from the Cancer Cell Line Encyclopedia (CCLE, DepMap, and 26Q1). Vertical lines indicate MITF expression levels for the four nonmelanocyte cancer cell lines selected for further analysis (HeLa, U2OS, A549, and SW1353). (B) Graphs representing mean of actual numbers of nuclear 53BP1 foci in 624mel, U2OS, A549, HeLa, D492, and SW1353 following treatment with the indicated siRNAs for 72 h and immunostaining with 53BP1 specific antibody. (C) Graphs representing proportion of cells with micronuclei in 624mel, U2OS, A549, HeLa, D492, and SW1353 following treatment with the indicated siRNAs for 72 h and staining with DAPI nuclear stain. Data presented as mean ± SD. *P < 0,05, **P < 0,01, ***P < 0,001, ****P < 0,0001.
**Fig. S2**. MITF knockdown causes genome instability in nonmelanocyte cell lines. 53BP1 foci distribution plots from the indicated cell lines following 48‐h treatment with control, MITF and BRCA2 targeting siRNAs. Each dot represents an individual cell, black horizontal line show the average number of foci from three independent experiments.
**Fig. S3**. MITF knockdown causes genome instability in nonmelanocyte cell lines. **A** BRCA2 mRNA expression in indicated cell lines. Cells were treated with siRNA targeting BRCA2 or siRNA control for 48 h, followed by real‐time qPCR analysis. (unpaired *t*‐test, *n* = 3). (B–C) Quantification of nuclear 53BP1 foci (F) and micronuclei (G) after indicated number of passages in a CRISPR/Cas9 MITF knockout U2OS cell line and an empty vector control cell line (unpaired *t*‐test, *n* = 3). (D) MITF mRNA expression in the CRISPR/Cas9 MITF knockout U2OS cell line and a U2OS cell line generated using empty vector (unpaired *t*‐test, *n* = 3). Data presented as mean ± SD. *P < 0,05, **P < 0,01, ***P < 0,001, ****P < 0,0001.
**Fig. S4**. MITF knockdown has an impact on genome stability, DNA replication and cell proliferation in U2OS cells. (A) Representative images of metaphase spreads for siCON (upper panel) and siMITF (lower panel) treated cells. Cells were treated with siRNA for 7 days. To enrich cells in metaphase, samples were treated with the mitotic inhibitor Colcemid 4 h prior to fixation. (B) Volcano plots showing number of genes affected by 48‐h siRNA mediated MITF knockdown in SkMel28 cells (upper panel) and U2OS cells (lower panel). Each dot in the volcano plot represents a gene affected by MITF knockdown in U2OS cells. Genes that are nonsignificantly affected are represented in black dots. (C) Quantification (right) and representative images (left) of Ki67 after 48‐h treatment with siMITF and siCON in U2OS cells. This was followed by immunostaining with a Ki67 antibody (unpaired *t*‐test, *n* = 3). Data presented as mean ± SD. *P < 0,05, **P < 0,01, ***P < 0,001, ****P < 0,0001.
**Fig. S5**. MITF knockdown in U2OS cells leads to replication stress and decreased invasion. (A) 53BP1 nuclear bodies after 48‐h siRNA treatment (siCON, siMITF, siBRCA1, and si53BP1), followed by immunostaining with antibodies targeting 53BP1 and CyclinA. Scale: 20 μm (Fig. 3B representative images) (one‐way ANOVA, *n* = 3). (B) 53BP1 nuclear bodies after 48‐h Doxycycline inducible MITF knockdown in 624mel cells, followed by immunostaining with antibodies targeting 53BP1 and CyclinA. Scale: 20 μm (Fig. 3C representative images) (one‐way ANOVA, *n* = 3). (C) 53BP1 nuclear bodies in a CRISPR/Cas9 MITF knockout U2OS cell line and a U2OS cell line generated using empty vector. Scale: 20 μm (Fig. 3D representative images) (unpaired *t*‐test, *n* = 3). (D) Quantification (left) and representative images (right) of MITF expression before and after 48 Doxycycline inducible MITF knockdown in 624mel cells. This was followed by immunostaining with a MITF antibody (one‐way ANOVA, *n* = 3). (E) Representative fluorescent microscope images showing invading cells after 48 h siRNA treatment (siCON and siMITF), followed by DAPI staining. Scale: 1000 μm. Data presented as mean ± SD. *P < 0,05, **P < 0,01, ***P < 0,001, ****P < 0,0001.
**Fig. S6**. P53 is activated upon MITF knockdown in U2OS cells. (A) MITF and P53 protein expression in 624mel cells after 48 h siRNA treatment (siCON and siMITF), analyzed by western blot of whole cell extracts using indicated antibodies. SMC1 was used as loading control. (B) MITF and P53 mRNA expression in 624mel cells. Cells were treated with indicated siRNAs for 48 h, followed by real‐time qPCR analysis (unpaired *t*‐test, *n* = 3). (C) Quantification (right) and representative images (left) of P53 protein expression before and after 48 h Doxycycline inducible MITF knockdown in 624 mel cells. This was followed by immunostaining with MITF and P53 antibodies (one‐way ANOVA, *n* = 3). Data presented as mean ± SD. *P < 0,05, **P < 0,01, ***P < 0,001, ****P < 0,0001.
**Fig. S7**. P53 is activated upon MITF knockdown in U2OS cells. (A) Cell cycle profiles and graphs of siCON and siMITF treated U2OS‐stable P53 knockout cells. Cells were fixed after 48 h siRNA treatment, followed by staining of DNA content with DAPI nuclear stain and flow cytometry analysis (unpaired *t*‐test, *n* = 3). B Graph shows negative correlation between the expression of MITF and P53 in sarcoma patient samples. Each dot on the graph represents MITF expression (y‐axis) and P53 expression (x‐axis) of single tumor. Values were extracted from the TCGA database. Data presented as mean ± SD. *P < 0,05, **P < 0,01, ***P < 0,001, ****P < 0,0001.
**Fig. S8**. P53 activation in MITF knockdown cells is dependent on the Hippo pathway kinase LATS2. (A) Quantification of P53 protein levels in U2OS cells after same experimental conditions as in Fig. 5A with a different LATS2 siRNA. Scale: 20 μm (one‐way ANOVA, n = 3–5). (B) Cell cycle profiles of siCON, siMITF, siLATS2, and siMITF + siLATS2 treated U2OS cells. Cells were fixed after 48 h siRNA treatment, followed by staining of DNA content with 7‐aminoactinomycin D (7AAD) and flow cytometry analysis (unpaired *t*‐test, *n* = 4). (C) Cell cycle profiles of siCON and siMITF treated 624mel cells. Cells were fixed after 48 h siRNA treatment, followed by staining of DNA content with 7‐aminoactinomycin D (7AAD) and flow cytometry analysis (unpaired *t*‐test, *n* = 3). (D) MITF and LATS2 mRNA expression in indicated cell lines. Cells were treated with siRNA targeting siMITF, siLATS2, siMITF + siLATS2 or siRNA control for 48 h, followed by real‐time qPCR analysis. (unpaired *t*‐test, *n* = 3). Data presented as mean ± SD. *P < 0,05, **P < 0,01, ***P < 0,001, ****P < 0,0001.
**Fig. S9**. P53 activation in MITF knockdown cells is dependent on the Hippo pathway kinase LATS2. (A) MITF and P53 mRNA expression in indicated cell lines. Cells were treated with siRNA targeting siMITF, P53, siMITF + siP53, or siRNA control for 48 h, followed by real‐time qPCR analysis. (unpaired *t*‐test, *n* = 3). (B) LATS2 protein expression in U2OS cells after 48 h siRNA treatment (siCON siLATS2 #1 and siLATS2 #2), analyzed by western blot of whole cell extracts using LATS2 antibody. Β‐actin was used as loading control. (C) Confocal microscopy images (left) and quantification (right) of P53 protein levels in 624mel cells 48‐h after treatment with siCON, siMITF, siLATS2, and siMITF + siLATS2, cells were fixed and immunostained with a P53 specific antibody and DAPI to visualize nuclear cells. Scale: 20 μm (one‐way ANOVA, *n* = 3). Data presented as mean ± SD. *P < 0,05, **P < 0,01, ***P < 0,001, ****P < 0,0001.
**Fig. S10**. Kaplan–Meier survival data generated using the xena browser. In each graph, MITF high expressing tumors are represented in red and MITF low expressing tumors are represented in blue (log‐rank test). Each graph represents survival data from different tumor types: (A) all tumor types, (B) sarcoma, (C) adrenocortical cancer, (D) metastatic melanoma, and (E) kidney clear cell carcinoma. TCGA datasets, *P*‐values, cutoff value between high and low RNA expression, and number of patients are shown on graphs. RSE norm_count was used for TCGA target GTEx samples and FPKM for the other samples. Kaplan–Meier survival analyses were generated using the UCSC Xena browser (https://xena.ucsc.edu/).
**Data S1**. Sanger sequencing data of isoform‐specific PCR products presented in Fig. 1F.
**Data S2**. qPCR primers used for qPCRs in the study.

## Data Availability

Flow cytometry data have been deposited in the Flow depository: http://flowrepository.org/id/RvFrVYTvPpVgLRpylPZjOj0zanKPIEzRCG7LUrZMTm5MQWkC0Xzt2aXukiZpFutV (ID: FR‐FCM‐Z7B8) and http://flowrepository.org/id/RvFrqyXBg2JWNta8wrUFC7dAdozDqQaOo50of0pveXDjvZn40UeKytWhR8HRwUuF (ID: FR‐FCM‐Z7CG). RNA‐seq data have been deposited in the Gene Expression Omnibus under accession number: GSE264653.
