# Supplementary material for: MITF maintains genome stability in nonmelanocyte lineages
Source: Mol Oncol. 2026 Jun 10;20(11):2839–59. doi: 10.1002/1878-0261.70273 (PMC13398355; doi:10.1002/1878-0261.70273)
Supplement: Supplementary file 1 [file MOL2-20-2839-s001.docx]

**C**

**B**

Figure S1

**A**

MITF knockdown causes genome instability in non-melanocyte cell lines. **A** MITF expression distribution across 1719 cancer cell lines from the Cancer Cell Line Encyclopedia (CCLE, DepMap 26Q1). Vertical lines indicate MITF expression levels for the four non-melanocyte cancer cell lines selected for further analysis (HeLa, U2OS, A549 an SW1353). **B** Graphs representing mean of actual numbers of nuclear 53BP1 foci in 624mel, U2OS, A549, HeLa, D492 and SW1353 following treatment with the indicated siRNAs for 72h and immunostaining with 53BP1 specific antibody. **C** Graphs representing proportion of cells with micronuclei in 624mel, U2OS, A549, HeLa, D492 and SW1353 following treatment with the indicated siRNAs for 72h and staining with DAPI nuclear stain. Data presented as mean ± SD. **P* <0,05, ***P* <0,01, ****P* <0,001, *****P* <0,0001.

Figure S2

MITF knockdown causes genome instability in non-melanocyte cell lines. 53BP1 foci distribution plots from the indicated cell lines following 48h treatment with control, MITF and BRCA2 targeting siRNAs. Each dot represents an individual cell, black horizontal line show the average number of foci from three independent experiments.

**A**

**B**

**C**

**D**

Figure S3

MITF knockdown causes genome instability in non-melanocyte cell lines. **A** BRCA2 mRNA expression in indicated cell lines. Cells were treated with siRNA targeting BRCA2 or siRNA control for 48h, followed by real time qPCR analysis. (unpaired t-test, n=3). **B-C** Quantification of nuclear 53BP1 foci (F) and micronuclei (G) after indicated number of passages in a CRISPR/Cas9 MITF knockout U2OS cell line and an empty vector control cell line (unpaired t-test, n=3). **D** MITF mRNA expression in the CRISPR/Cas9 MITF knockout U2OS cell line and a U2OS cell line generated using empty vector (unpaired t-test, n=3). Data presented as mean ± SD. **P* <0,05, ***P* <0,01, ****P* <0,001, *****P* <0,0001.

Figure S4

MITF knockdown has an impact on genome stability, DNA replication and cell proliferation in U2OS cells. **A** Representative images of metaphase spreads for siCON (upper panel) and siMITF (lower panel) treated cells. Cells were treated with siRNA for seven days. To enrich cells in metaphase, samples were treated with the mitotic inhibitor Colcemid four hours prior to fixation. **B** Volcano plots showing number of genes affected by 48h siRNA mediated MITF knockdown in SkMel28 cells (upper panel) and U2OS cells (lower panel). Each dot in the volcano plot represents a gene affected by MITF knockdown in U2OS cells. Genes that are non-significantly affected are represented in black dots. **C** Quantification (right) and representative images (left) of Ki67 after 48h treatment with siMITF and siCON in U2OS cells. This was followed by immunostaining with a Ki67 antibody (un-paired t-test, n=3). Data presented as mean ± SD. **P* <0,05, ***P* <0,01, ****P* <0,001, *****P* <0,0001.

Figure S5

MITF knockdown in U2OS cells leads to replication stress and decreased invasion. **A** 53BP1 nuclear bodies after 48h siRNA treatment (siCON, siMITF, siBRCA1, si53BP1), followed by immunostaining with antibodies targeting 53BP1 and CyclinA. Scale: 20μM (Figure 3B representative images) (one-way-ANOVA, n=3). **B** 53BP1 nuclear bodies after 48h Doxycycline inducible MITF knockdown in 624mel cells, followed by immunostaining with antibodies targeting 53BP1 and CyclinA. Scale: 20μm (Figure 3C representative images) (one-way-ANOVA, n=3). **C** 53BP1 nuclear bodies in a CRISPR/Cas9 MITF knockout U2OS cell line and a U2OS cell line generated using empty vector. Scale: 20μm (Figure 3D representative images) (unpaired t-test, n=3). **D** Quantification (left) and representative images (right) of MITF expression before and after 48 Doxycycline inducible MITF knockdown in 624mel cells. This was followed by immunostaining with a MITF antibody (one-way ANOVA, n=3). **E** Representative fluorescent microscope images showing invading cells after 48h siRNA treatment (siCON and siMITF), followed by DAPI staining. Scale: 1000μM. Data presented as mean ± SD. **P* <0,05, ***P* <0,01, ****P* <0,001, *****P* <0,0001.

+

-

siMITF

Doxocyclin

Untreated

miCON

miMITF

**MITF p53**

**C**

**A**

**B**

MITF

SMC1

p53

55

kDa

55

180

Figure S6

P53 is activated upon MITF knockdown in U2OS cells. **A** MITF and P53 protein expression in 624mel cells after 48h siRNA treatment (siCON and siMITF), analyzed by western blot of whole cell extracts using indicated antibodies. SMC1 was used as loading control. **B** MITF and P53 mRNA expression in 624mel cells. Cells were treated with indicated siRNAs for 48h, followed by real time qPCR analysis (unpaired t-test, n=3). **C** Quantification (right) and representative images (left) of P53 protein expression before and after 48h Doxycycline inducible MITF knockdown in 624mel cells. This was followed by immunostaining with MITF and P53 antibodies (one-way ANOVA, n=3). Data presented as mean ± SD. **P* <0,05, ***P* <0,01, ****P* <0,001, *****P* <0,0001.

DNA content

Count

**A**

**B**

Figure S7

P53 is activated upon MITF knockdown in U2OS cells. **A** Cell cycle profiles and graphs of siCON and siMITF treated U2OS-stable P53 knockout cells. Cells were fixed after 48h siRNA treatment, followed by staining of DNA content with DAPI nuclear stain and flow cytometry analysis (unpaired t-test, n=3). **B** Graph shows negative correlation between the expression of MITF and P53 in sarcoma patient samples. Each dot on the graph represents MITF expression (y-axis) and P53 expression (x-axis) of single tumor. Values were extracted from the TCGA database. Data presented as mean ± SD. **P* <0,05, ***P* <0,01, ****P* <0,001, *****P* <0,0001.

Counts

**D**

**B**

DNA content

**C**

Count

DNA content

**A**

Figure S8

P53 activation in MITF knockdown cells is dependent on the Hippo pathway kinase LATS2. **A** Quantification of P53 protein levels in U2OS cells after same experimental conditions as in Figure 5A with a different LATS2 siRNA. Scale: 20μM (one-way-ANOVA, n=3-5). **B** Cell cycle profiles of siCON, siMITF, siLATS2 and siMITF + siLATS2 treated U2OS cells. Cells were fixed after 48h siRNA treatment, followed by staining of DNA content with 7-aminoactinomycin D (7AAD) and flow cytometry analysis (unpaired t-test, n=4). **C** Cell cycle profiles of siCON and siMITF treated 624mel cells. Cells were fixed after 48h siRNA treatment, followed by staining of DNA content with 7-aminoactinomycin D (7AAD) and flow cytometry analysis (unpaired t-test, n=3). **D** MITF and LATS2 mRNA expression in indicated cell lines. Cells were treated with siRNA targeting siMITF, siLATS2, siMITF + siLATS2 or siRNA control for 48h, followed by real time qPCR analysis. (unpaired t-test, n=3). Data presented as mean ± SD. **P* <0,05, ***P* <0,01, ****P* <0,001, *****P* <0,0001.

P53

DAPI

siCON

siMITF

siLATS2

siMITF_LATS2

LATS2 siRNA

LATS2

B-actin

-

#1

#2

#2

#1

-

kDa

150

45

**C**

**B**

**A**

Figure S9

P53 activation in MITF knockdown cells is dependent on the Hippo pathway kinase LATS2. **A** MITF and P53 mRNA expression in indicated cell lines. Cells were treated with siRNA targeting siMITF, P53, siMITF + siP53 or siRNA control for 48h, followed by real time qPCR analysis. (unpaired t-test, n=3). **B** LATS2 protein expression in U2OS cells after 48h siRNA treatment (siCON siLATS2 #1 and siLATS2 #2), analyzed by western blot of whole cell extracts using LATS2 antibody. Β-actin was used as loading control. **C** Confocal microscopy images (left) and quantification (right) of P53 protein levels in 624mel cells 48h after treatment with siCON, siMITF, siLATS2 and siMITF + siLATS2, cells were fixed and immunostained with a P53 specific antibody and DAPI to visualize nuclear cells. Scale: 20μM (one-way-ANOVA, n=3). Data presented as mean ± SD. **P* <0,05, ***P* <0,01, ****P* <0,001, *****P* <0,0001.

Figure S10

Kaplan-Meier survival data generated using xena browser. In each graph MITF high expressing tumors are represented in red and MITF low expressing tumors are represented in blue (log-rank test). Each graph represents survival data from different tumor types: **A** all tumor types, **B** sarcoma, **C** adrenocortical cancer, **D** metastatic melanoma and **E** kidney clear cell carcinoma. TCGA datasets, p-values, cut-off value between high and low RNA expression, and number of patients are shown on graphs. RSE norm_count was used for TCGA target GTEx samples and FPKM for the other samples. Kaplan-Meier survival analyses were generated using the UCSC Xena browser (<https://xena.ucsc.edu/>).

**Supplementary data 1**

Sanger sequencing data of isoform specific PCR products presented in Figure 1F.

**U2OS cells, A-isoform 1**

AGTACGCTGA GAGCAGTTGG CCTGGTCTCG GGACTTGATT GATAAGCCTC CGATAACCTC	61 CTCCAGTATG ACATCACGCA TCTTGCTACG CCAACAACTC ATGGGTGAAC AGATGCTGGA 121 CCAGGATCGC AGGGAGCAGC AGCAGAAGCT GCAGGCGGCC CAATTCATGC AACAAAGAGT 181 GCCCGTGAGT CAAACACCAT CCATAAGCGT CAGTGTGGCC ACCACCCTTC CCTCTGCCTC 241 GCAGGGGCCG ATGGAAGTCC TGAAGGTGCA AACCCACCTC AAAAACCCCA CCAAGTACCA 301 CATACAGCAA GCCCAACGGC AGCAGGTAAA GCAGTACCTT TCTACCACTT TATCAAATAA 361 ACATGCCAAC CAAGTCCTGA GCTTGCCATG TCCAAACCAG CCTGGCGATC ATGTCATGCC 421 ACCGGTGCCG GTCAGCAGCG CACCCAACAG CCACATGGCT ATGCTTACGC TTAACTCCAA 481 CTGTGAAAAA GAGGGATTTT ATAAGTTTGA AGAGCAAAAC AGGGCAGAGA GCGAGTGCCC 541 AGGCATGAAC ACACATTCAC	GAGCGTCCTG	TATGCAGATG GATGATGTAA TCGATGACAT	601 CATTAGCCTA GAATCAAGTT ATAATGAGGA AATCTTGGGC TTGATGGATC CTGCTTTGCA	661 AATGGCAAAT	ACGTTGCCTG TCTCGGGAAA TTGGATTTGA TCA //

**SkMel28 cells, A-isoform 1**

ATGTACGCTG AGAGCAGTTG CCTGTCTCGG GAACTTGATT GATCAGCCTC CGATAAGCTC	61 CTCCAGTATG ACATCACGCA TCTTGCTACG CCAGCAACTC ATGCGTGAGC AGATGCAGGA 121 GCAGGAGCGC ATGGAGCAGC AGCAGAAGCT GCAGGCGGCC CAGTTCATGC AACAGAGAGT 181 GCCCGTGAGT CAGACACCAG CCATAAACGT CAGTGTGCCC ACCACCCTTC CCTCTGCCAC 241 GCAGGTGCCG ATGGAAGTCC TTAAGGTGCA GACCCACCTC GAAAACCCCA CCAAGTACCA 301 CATACAGCAA GCCCAACGGC AGCAGGTAAA GCAGTACCTT TCTACCACTT TAGCAAATAA 361 ACATGCCAAC CAAGTCCTGA GCTTGCCATG TCCAAACCAG CCTGGCGATC ATGTCATGCC 421 ACCGGTGCCG GGGAGCAGCG CACCCAACAG CCCCATGGCT ATGCTTACGC TTAACTCCAA 481 CTGTGAAAAA GAGGGATTTT ATAAGTTTGA AGAGCAAAAC AGGGCAGAGA GCGAGTGCCC 541 AGGCATGAAC ACACATTCAC GAGCGTCCTG TATGCAGATG	GATGATGTAA	TCGATGACAT	601

CATTAGCCTA	GAATCAAGTT ATAATGAGGA AATCTTGGGC TTGATGGATC CTGCTTTGCA	661

AATGGCAAAT ACGTTGCCTG TCTCGGGAAA CTTGATTGAT CA //

**SkMel28 cells, M-isoform 1**

TGGGAGGGAT AGTCTACCGT CTCTCACTGG GATTGGTGCC	ACCTAAAACA	TTGTTATGCT	61

GGAAATGCTA GAATATAATC ACTATCAGGT GCAGACCCAC CTCGAAAACC CCACCAAGTA 121 CCACATACAG CAAGCCCAAC GGCAGCAGGT AAAGCAGTAC CTTTCTACCA CTTTAGCAAA 181 TAAACATGCC AACCAAGTCC TGAGCTTGCC ATGTCCAAAC CAGCCTGGCG ATCATGTCAT 241 GCCACCGGTG CCGGGGAGCA GCGCACCCAA CAGCCCCATG GCTATGCTTA CGCTTAACTC 301 CAACTGTGAA AAAGAGGGAT TTTATAAGTT TGAAGAGCAA AACAGGCAGA GAGCGAGTGC 361 CCAGGCATGA ACACACATTC ACGAGCGTCC TGTATGCAGA	TGGATGATGT AATCGATGAC	421 ATCATTAGCC	TAGAATCAAG TTATAATGAG GAAATCTTGG GCTTGATGGA TCCTGCTTTG	481

CAAATGGCAA	ATACGTTGCC TGTCTCGGGA	ACTGCATTTG ATAAA //

**Supplementary data 2**

qPCR primers used for qPCRs in the study.

| *MITF* | Forward _TTCCCACAGAGTCTGAAGCAAG_ |
| --- | --- |
|  | Reverse _TCCAGCGCATGTCTGGATCA_ |
| *P53* | Forward _ CTTCCCTGGATTGGCAGC_ |
|  | Reverse _ TTTCAGGAAGTAGTTTCCATAGGT_ |
| *LATS2* | Forward _CAGGATGCGACCAGGAGATG_ |
|  | Reverse _AGGTCTGCTTAATGACCCGC_ |
| MITF - isoform A specific | Forward _TGAAGAGCCCAAAACCTATTACGA_ |
|  | Reverse _GATCAATCAAGTTTCCCGAGACAG_ |
| MITF - isoform M specific | Forward _CCTTCTCTTTGCCAGTCCATCTTC_ |
|  | Reverse _GATCAATCAAGTTTCCCGAGACAG_ |
| MITF - isoform C specific | Forward _CTTCAGTGGTTTTCCCACGAGCT_ |
|  | Reverse _GATCAATCAAGTTTCCCGAGACAG_ |
| MITF - isoform H specific | Forward _GGAGGCGCTTAGAGTTCAGATG_ |
|  | Reverse _GATCAATCAAGTTTCCCGAGACAG_ |
